# The "Respectability" of the Out-Patient

**Published:** 1906-09-08

**Authors:** 


### THE "RESPECTABILITY" OF THE OUT-PATIENT.

It is daily becoming more and more respectable to attend the out-patient department of a hospital. The ever-growing incubus of out-patients has long been a serious problem in hospital management. The service of the out-patient departments is in general so good, and the means of controlling admissions to them are so difficult of application, that some degree of abuse is almost inevitable. No one can read without surprise and misgiving the out-patient returns of hospitals both large and small. The London Hospital, for instance, the largest of the Metropolitan charities, dealt in the course of 1904 with no fewer than 206,000 out-patients. The prospectus from which these figures are taken does not specify whether they refer to new patients only or to attendances, but even a 50 per cent. error would hardly affect the issue. We have to face the fact that the figures of out-patient practice in London are colossal, and that, by a curious irony, the heaping-up of out-patients is accounted to a hospital for righteousness, and stands in many quarters as an index of efficiency and merit.

It is well, in view of these circumstances, to clear our minds as to the elementary functions of the out-patient service of a public hospital. It is easy to define these functions academically. The out-patient service is intended for urgent accidents of all kinds, and among all classes, and for the treatment of those who require treatment and are yet too poor to pay for private medical attendance. Departure from this premiss, therefore, constitutes hospital abuse. It is easy to placard the walls of an out-patient room with notices to the effect that trivial ailments will not be treated, but a very brief practical experience of the conditions of out-patient practice will suffice to demonstrate the impossibility of observing such a rule. Although the instinct of the overtaxed medical officer may assure him that such and such a case is trifling and not deserving of relief at the public charges, he wisely will not risk his professional reputation to prevent an abuse which only the most searching and detailed investigation would enable him to establish with certainty. It is in this particular that the free, or practically free, medical service of out-patient departments conspicuously fails. There can be no question that thousands of patients are yearly treated at hospitals for disorders so trifling that middle-class people who pay would, under similar circumstances, never think of incurring a doctor's bill on their account. It is equally certain that a considerable proportion of these trivial disorders, in addition to a vast number of graver ones, are directly attributable to the abuse of alcohol. It is impossible to resist the conclusion that the publican and the tobacconist grow rich at the expense of the public charities and of the private doctor.

These strictures must not be taken as an indictment of the out-patient system as a whole, for such abuses as exist fall more upon the executive hospital staffs and private practitioners than upon the purse of charity. The medical officers of out-patient and casualty departments are drawn either from the ranks of young consultants or from the recently qualified members of the profession, men of the standing of house physicians and house surgeons. To both these classes experience is the prime necessity of the moment. Hence they are content to assume, for a purely nominal monetary return, labours whose extent is little appreciated by the public. It is no great rarity for one man to deal personally with a hundred new cases in the course of the morning, a state of affairs which is only compatible with the general success of out-patient departments on the assumption that a great preponderance of the applicants have only trifling disorders. None the less the great burden of work militates seriously against the value of such posts to their possessors, and none would view a reduction in out-patients with greater gratification than the staffs of hospitals. Our hospital system provides a service of immense beneficence and utility, but in the matter of the out-patient departments there is room for wise reforms. We cannot in this brief space even adumbrate the various schemes for betterment which have been propounded from time to time. It is, however, essentially fallacious to estimate the efficient charity of a hospital by the number of its out-patients. The efficiency of a hospital charity should rather be estimated in the inverse ratio of the crowds in its out-patient department.

